# Cellular Physiology and Pathophysiology of EAAT Anion Channels

**DOI:** 10.3389/fncel.2021.815279

**Published:** 2022-01-06

**Authors:** Peter Kovermann, Miriam Engels, Frank Müller, Christoph Fahlke

**Affiliations:** Institute of Biological Information Processing, Molekular- und Zellphysiologie (IBI-1), Forschungszentrum Jülich, Jülich, Germany

**Keywords:** excitatory amino acid transporter, glutamate transporter, anion channels, chloride homeostasis, retina, channelopathies

## Abstract

Excitatory amino acid transporters (EAATs) optimize the temporal resolution and energy demand of mammalian excitatory synapses by quickly removing glutamate from the synaptic cleft into surrounding neuronal and glial cells and ensuring low resting glutamate concentrations. In addition to secondary active glutamate transport, EAATs also function as anion channels. The channel function of these transporters is conserved in all homologs ranging from archaebacteria to mammals; however, its physiological roles are insufficiently understood. There are five human EAATs, which differ in their glutamate transport rates. Until recently the high-capacity transporters EAAT1, EAAT2, and EAAT3 were believed to conduct only negligible anion currents, with no obvious function in cell physiology. In contrast, the low-capacity glutamate transporters EAAT4 and EAAT5 are thought to regulate neuronal signaling as glutamate-gated channels. In recent years, new experimental approaches and novel animal models, together with the discovery of a human genetic disease caused by gain-of-function mutations in EAAT anion channels have enabled identification of the first physiological and pathophysiological roles of EAAT anion channels.

## Introduction

Glial and neuronal excitatory amino acid transporters (EAATs) ensure low resting neurotransmitter concentrations in the synaptic cleft and prevent glutamate excitotoxicity by transporting glutamate from the synaptic cleft into neuronal and glial cells (Danbolt, 2001; Kanner, 2006). EAATs are prototypical dual function proteins that act as both secondary active glutamate transporters and anion channels (Fairman et al., 1995; Wadiche et al., 1995; Larsson et al., 1996). Whereas, the molecular basis of these two transport functions is now well-understood (Fahlke et al., 2016), we are just starting to appreciate the cellular functions of these glutamate-gated chloride channels in the human body.

About 30 years ago, EAAT anion channel behavior was discovered almost simultaneously in two different experimental systems: cloned transporters in heterologous expression systems and native transporters in retinal preparations. Heterologous expression of EAAT1, EAAT2, and EAAT3 in *Xenopus* oocytes and electrophysiological analysis revealed the existence of a current component carried by anions that is not coupled to electrogenic glutamate transport (Wadiche et al., 1995). Moreover, after identification and characterization of the first three isoforms (Kanai and Hediger, 1992; Pines et al., 1992; Storck et al., 1992), homology cloning of EAAT4 (Fairman et al., 1995) and EAAT5 (Arriza et al., 1997) revealed the existence of family members that predominantly function as anion channels.

A glutamate-gated anion channel with many of the functional features of EAAT glutamate transporters was first observed on cone photoreceptors of salamander retina (Picaud et al., 1995b; Larsson et al., 1996) and on dendrites of ON-bipolar cells in white perch retina (Grant and Dowling, 1995; Picaud et al., 1995b; Larsson et al., 1996). On cone photoreceptors, this channel allows cells to respond to glutamate that they themselves have released and provides a feedback signal about the concentration of glutamate released into the synaptic cleft (Picaud et al., 1995a). In ON-bipolar cells of white perch retina, chloride conductance mediates an inhibitory input that keeps the cells hyperpolarized in the dark, when photoreceptors release glutamate. The retina-specific mammalian EAAT5 (Arriza et al., 1997; Gameiro et al., 2011; Schneider et al., 2014) closely resembles salamander *s*EAAT5A (Eliasof et al., 1998) and is, thus, assumed to fulfill similar functions in the mammalian retina. However, until recently, the lack of EAAT5-specific blockers and an EAAT5-knockout animal model have prevented the experimental verification of this hypothesis.

Here we review recent progress in the cellular physiology and pathophysiology of EAAT anion channels.

## EAAT Anion Channels Exhibit Low Unitary Current Amplitudes and Absolute Open Probabilities

**Figure 1:**
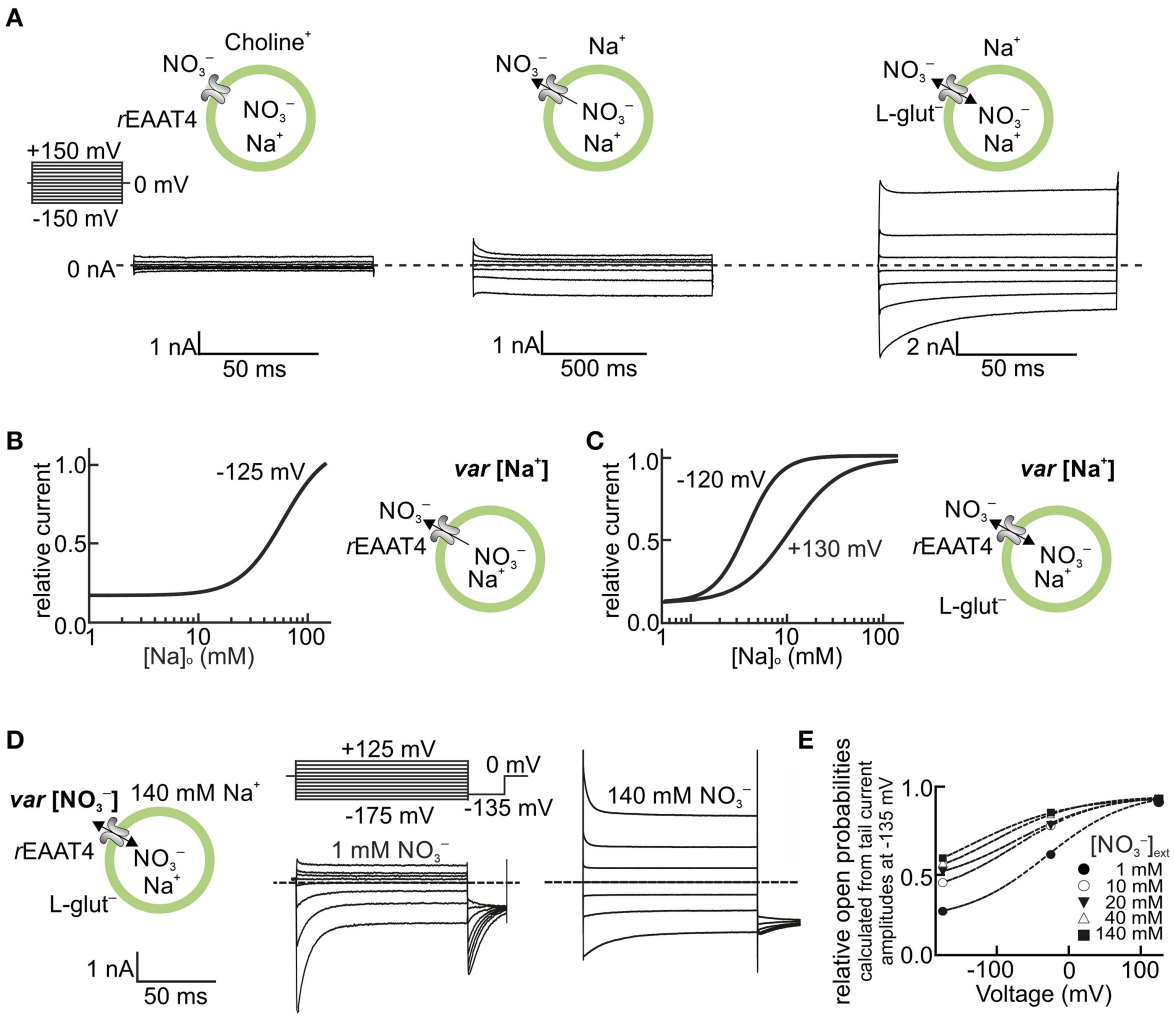
EAAT anion channels are modulated by transport substrates and permeant anions. **(A–C)** Na^+^ dependence of EAAT4 anion channels heterologously expressed in HEK293T cells. **(A)** Representative whole-cell current recordings of rat EAAT4 with symmetric ${\text{NO}}_{3}^{+}$ as the permeable anion, in the absence of external Na^+^ and L-glutamate (Glu^−^) (left), in the presence of external Na^+^ (middle), and in the presence of external Na^+^ and 5 mM L-glutamate (right, see insets). The Na^+^ concentration dependence of EAAT4 anion channel currents is shown in the absence **(B)** and presence **(C)** of external L-glutamate for high positive and negative voltages (internal Na^+^ concentration in **A,B** was set to ~114 mM). **(D,E)** Dependence of EAAT4 anion channel gating on permeant anions. **(D)** Representative whole-cell recordings from rat EAAT4 whole-cell currents with different external concentrations of ${\text{NO}}_{3}^{+}$ (1–140 mM) (see insets, the internal ${\text{NO}}_{3}^{+}$ concentration in **C,D** was set to 110 mM). **(E)** Relative open probabilities of rat EAAT4 anion channels at different external concentrations of the permeant anion ${\text{NO}}_{3}^{+}$. In the depicted experiments, Na^+^ was replaced by equimolar amounts of choline **(A–C)**, and ${\text{NO}}_{3}^{+}$ by the impermeant anion D-gluconate^−^ **(D,E)**. Relative open probabilities in E were calculated from instantaneous tail current amplitudes at −135 mV, as shown in the voltage protocol in D. rEAAT4: rat EAAT4. var, varying. This figure is modified and reprinted from Kovermann et al. (2010), with permission.

Initially, EAAT anion channels were mainly studied in *Xenopus* oocytes. In this system, EAAT anion currents are small compared with endogenous current components and subtraction procedures are usually needed to identify the EAAT anion channel-specific current (Fairman et al., 1995; Wadiche et al., 1995; Wadiche and Kavanaugh, 1998; Ryan et al., 2004; Cater et al., 2014, 2016). The use of a mammalian cell expression system and more permeable anions permit the direct recording of EAAT anion currents under various conditions. Figure 1 shows representative EAAT4 anion current recordings from HEK293T cells expressing rat EAAT4. Because of the current convention, inward currents at negative potentials correspond to anion efflux. The currents are small in the absence of Na^+^ and glutamate, and the application of Na^+^ alone or of Na^+^ plus glutamate increases current amplitudes in a dose-dependent manner. EAAT4 anion currents exhibit time- and voltage-dependent changes in current amplitudes that resemble voltage-dependent gating (Figures 1A–C) (Kovermann et al., 2010). This “gating” is affected by transporter substrates (Machtens et al., 2011), but also by permeant anion concentrations (Figures 1D,E) (Kovermann et al., 2010). Such experiments support the notion that EAAT anion channels need Na^+^ ions to be active (Mim et al., 2005; Tao et al., 2006; Grewer et al., 2008). However, glial EAAT anion channels are Na^+^ independent in the absence of glutamate (Leinenweber et al., 2011; Divito et al., 2017) and can also be active when K^+^ is the only monovalent cation present (Kortzak et al., 2019).

Since EAAT anion currents are large compared with uptake currents and are not coupled to glutamate transport, they were always assumed to be channel mediated. Definitive proof was provided by measurements of unitary current amplitudes *via* noise analysis. Larsson et al. (1996) studied glutamate transporter-associated anion channels on tiger salamander cones using whole-cell patch-clamp recordings. They demonstrated that glutamate transporter-associated current fluctuations are Lorentzian, i.e., generated by the random opening and closing of individual channels. Stationary noise analysis involving the adjustment of anion channel open probabilities by modifying external glutamate provided a single-channel conductance of 0.7 pS at symmetric chloride concentration [Cl^−^] (Picaud et al., 1995b; Larsson et al., 1996). These single-channel amplitudes are too high to be accounted for by carrier-mediated transport. Thus, these data establish channel-like anion conduction by EAAT glutamate transporters.

Noise analysis of mammalian EAAT1–EAAT5 proteins in transfected HEK293T cells provided a unitary conductance of around 1 pS: EAAT5 had the highest single-channel amplitude (Schneider et al., 2014), EAAT4 (Torres-Salazar and Fahlke, 2007) had the lowest, and the high-capacity transporters EAAT1 (Winter et al., 2012), EAAT2 (Schneider et al., 2014), and EAAT3 (Torres-Salazar and Fahlke, 2007) displayed intermediate values. Noise analysis of the EAAT-associated anion channel amplitudes demonstrated identical unitary current amplitudes for various external [glutamate], indicating the existence of a single anion pore that is either open or closed (Kovermann et al., 2010). Absolute open probabilities (determined by comparing EAAT anion and transport currents) turned out to be extremely low, i.e., 0.06 ± 0.01% for EAAT2 (Kolen et al., 2020).

## Molecular Determinants of EAAT-Associated Anion Channel Function

EAATs assemble as homo- or heterotrimers (Gendreau et al., 2004; Yernool et al., 2004; Nothmann et al., 2011), with each subunit mediating both transport functions independently of its neighboring subunits (Grewer et al., 2005; Koch et al., 2007; Leary et al., 2007). They are prototypical elevator transporters: each subunit contains a trimerization domain that provides a scaffold for transmembrane movements of the transport domain and has binding sites for all substrates (Yernool et al., 2003; Boudker et al., 2007; Reyes et al., 2009; Verdon and Boudker, 2012; Jensen et al., 2013; Verdon et al., 2014; Guskov et al., 2016; Arkhipova et al., 2019). Substrate transport is based on large-scale (~18 Å) rotational translational movement of the transport domain relative to the static trimerization domain (Crisman et al., 2009; Reyes et al., 2009). For many years, none of the reported structures exhibited a hydrophilic pore-like structure that could be structurally correlated to the EAAT anion pore.

A convincing molecular model of the EAAT anion pore was obtained *via* molecular dynamics simulation of Glt_Ph_ (Machtens et al., 2015). At the time, Glt_Ph_ structures were available for the inward- (Reyes et al., 2009) and outward-facing conformations (Yernool et al., 2004; Boudker et al., 2007; Verdon et al., 2014), as well as for one intermediate state (Verdon and Boudker, 2012). Each of these conformations was tested for possible anion permeation using a computational electrophysiology approach that permitted the observation of ion permeation events under constant voltages (Kutzner et al., 2011). At a [NaCl] of 1 M and voltages of around 800 mV, no Cl^−^ permeation event was observed for any of these conformations. However, in simulations starting from various intermediate conformations—either using a published structure (Verdon and Boudker, 2012) or obtained using an essential dynamics approach—lateral movement of the transport domain opened the interface between the trimerization and transport domains. Subsequent wetting caused the formation of an anion-selective pore with functional properties resembling experimental results for EAAT anion channels.

This novel conformation (named ChC) was experimentally verified using tryptophan-scanning mutagenesis and by combined *in silico* and *in vitro* mutagenesis. Tryptophan fluorescence is collisionally quenched by anions; thus, fluorescence spectroscopy at various anion concentrations permits tryptophan side chains protruding onto the anion permeation pathway to be distinguished from those in other localizations. Predictions of side-chain accessibility to the aqueous medium in different conformations, including the ChC conformation, perfectly matched with iodide (I^−^) accessibility for Glt_Ph_ mutants with a single tryptophan substitution (Machtens et al., 2015). Computational electrophysiology is well-suited to estimate the effects of amino acid exchange on unitary current amplitudes and anion-to-cation selectivity. An extensive mutational scan revealed perfect agreement between simulated permeation properties and experimental values, which included unitary current amplitudes determined by noise analysis and anion-cation selectivities obtained from whole-cell recordings under varying anion/cation gradients. These results demonstrate that the ChC conformation is indeed formed and is responsible for anion conduction in EAATs (Machtens et al., 2015).

Recently, an intermediate conformation of Glt_Ph_, captured *via* crosslinking of an inserted cysteine pair was obtained by cryo-electron microscopy (Chen et al., 2021). The authors used molecular dynamics simulation to show the formation of a continuous hydration pathway at the interface of transport and trimerization domain. Subsequently, umbrella sampling simulations were used to determine free-energy profiles for pulling Cl^−^ through this aqueous pore. However, no spontaneous permeations were reported, and it is therefore not clear whether this intermediate conformation can conduct anions under physiological chemical/electrical gradients without application of external force. Neither unitary currents nor selectivities between anions or between anions and cation were computed. To experimentally support the role of the novel intermediate conformation in EAAT anion permeation, EAAT1 was mutated at seven position (Chen et al., 2021; #4525). Most of these mutations were already tested by Machtens et al. (2015). Each of the mutation modified reversal potentials of combined EAAT1 transport/anion currents. This analysis does not permit to separate effects of the mutations on glutamate transport, on the likelihood of anion channel opening or on the unitary current amplitude. All tested mutations will also affect the ChC anion conduction pathway, and the used crosslink does not prevent formation of the ChC conformation. Thus, neither experiments nor simulations demonstrate that the novel intermediate conformation is anion-conducting.

At present, it is therefore not possible to assess the functional role of this intermediate conformation. It may represent an additional anion-conductive state under physiological conditions or a translocation intermediate, from which anion channel opening may occur. In contrast, the ChC conformation has been shown to account for EAAT anion conduction with permeation rates and selectivity in full agreement with experimental results and to explain all available mutagenesis results (Machtens et al., 2015).

## EAAT Anion Channels Contribute to Chloride Homeostasis in Glial Cells

The association of a missense mutation in *SLC1A3* (encoding the glial glutamate transporter EAAT1) with a case of a human genetic disease (Jen et al., 2005; Winter et al., 2012) was the basis to evaluate the role of EAAT anion channels in glial chloride homeostasis. Glial cells display predominant K^+^ conductance that allows the buffering of [K^+^] in the extracellular space and ensures a stable negative resting potential (Lothman and Somjen, 1975; Futamachi and Pedley, 1976). The K^+^ conductance prevents glial depolarization upon electrogenic glutamate uptake. However, internal Cl^−^ concentrations ([Cl^−^]_int_) in glial cells are larger than expected from passive distribution (Kimelberg, 1981; Kettenmann et al., 1987; Bevensee et al., 1997; Walz, 2002), and EAAT anion channels might reduce the resting [Cl^−^]_int_ *via* mediating a chloride efflux pathway in parallel to K^+^ efflux through glial K^+^ channels.

**Figure 2:**
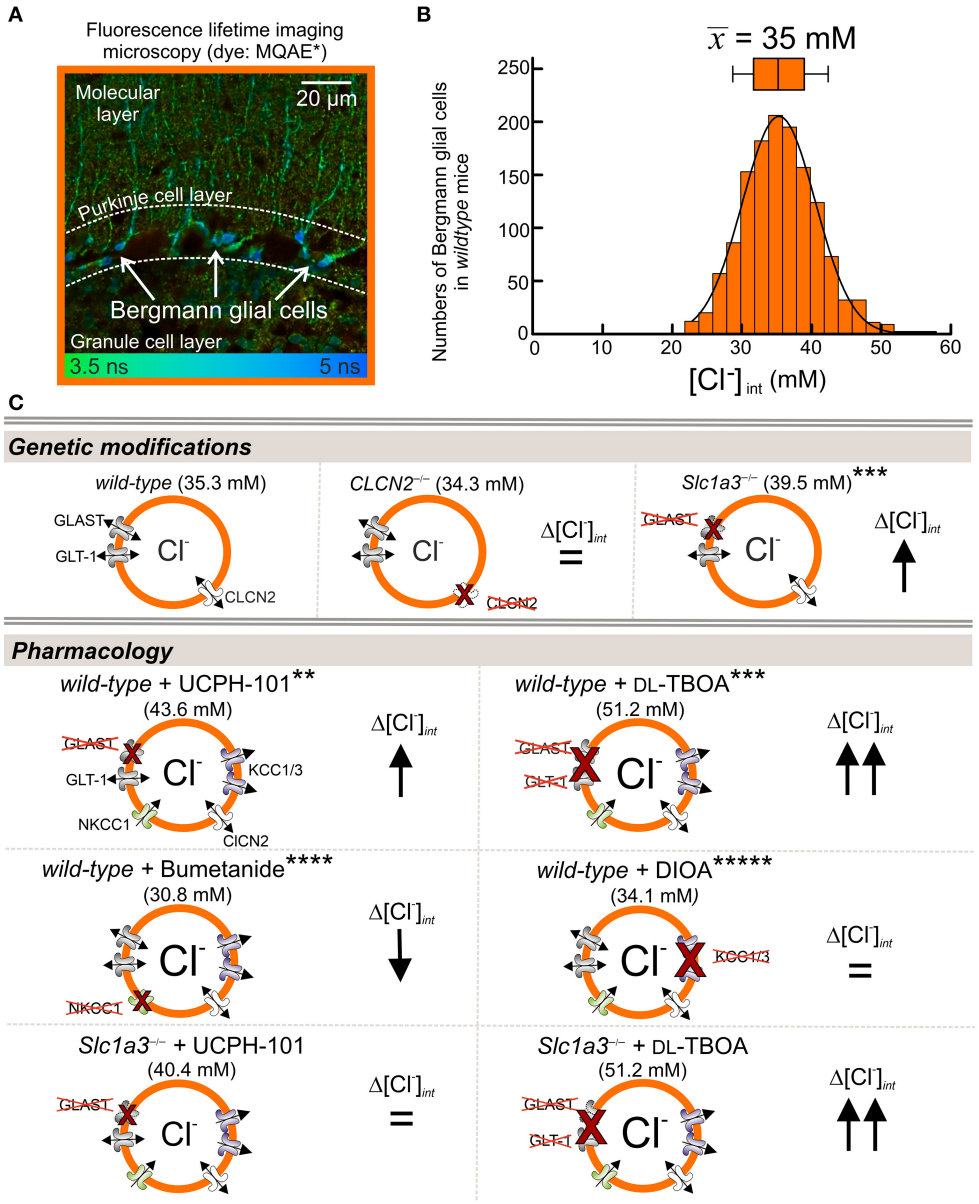
EAAT1/GLAST contributes to [Cl^−^]_int_ in Bergmann glial cells. **(A,B)** Fluorescence lifetime imaging with the Cl^−^-sensitive dye MQAE. **(A)** Representative FLIM images from acute sagittal slices of the cerebellar cortex show MQAE-stained Bergmann glial cells (BGLs, arrows) in the Purkinje cell layer. **(B)** Distribution of [Cl^−^]_int_ from BGLs, with a mean (± SD) of 35.3 ± 6.3 mM. **(C)** [Cl^−^]_int_ is higher in GLAST-knockout (*Slc*1*a*3^−/−^) than in wild-type (WT) BGLs, but is not affected by genetic ablation of CLCN2 (chloride voltage-gated channel 2). [Cl^−^]_int_ increases upon pharmacological blockage by the EAAT1-specific inhibitor UCPH-101 and the EAAT-specific blocker dl-TBOA. Reduction of Cl^−^ import by the NKCC1-specific inhibitor bumetanide decreases [Cl^−^]_int_, whereas inhibition of glial Cl^−^-exporters KCC1 and KCC3 by DIOA has no effect on [Cl^−^]_int_. Control experiments with *Slc*1*a*3^−/−^ mice showed, that GLT-1 also contributes to chloride homeostasis (lower panel). Modified and reprinted from Untiet et al. (2017), with permission. *MQAE: 6-(Methoxychinolinio)acetic acid ethyl ester bromide (C_14_H_16_BrNO_3_). **UCPH-101: 2-Amino-4-(4-methoxyphenyl)-7-(1-naphthyl)-5-oxo-5,6,7,8-tetrahydro-4H-chromene-3-carbonitrile (C_27_H_22_N_2_O_3_). ***DL-TBOA: DL-Threo-β-benzyloxyaspartate (C_11_H_13_NO_5_). ****Bumetanide: 3-Butylamino-4-phenoxy-5-sulfamoyl benzoic acid (C_17_H_20_N_2_O_5_S). *****DIOA: R(+)-Butylindazone (C_20_H_24_Cl_2_O_4_).

Untiet et al. (2017) measured [Cl^−^]_int_ in Bergmann glial cells in acute cerebellar slices using fluorescence lifetime imaging (FLIM) with the Cl^−^-sensitive dye MQAE. The experiments revealed a mean resting concentration of 35 mM in juvenile animals (postnatal days P20–P30; Figures 2A,B). Blocking the cation–chloride cotransporter NKCC1 decreased [Cl^−^]_int_ in Bergmann glial cells, whereas DIOA inhibition of two glial K^+^-Cl^−^ cotransporters, KCC1 and KCC3 (Figure 2C), had only minor effects on [Cl^−^]_int_. In *Slc1a3*^−/−^ mice lacking the glutamate transporter EAAT1/GLAST, but not in *Clc2*^−/−^ mice, [Cl^−^]_int_ was increased to 40 mM (Figure 2C). Inhibition of EAAT1/GLAST by the highly specific EAAT1 blocker UCPH-101 (Abrahamsen et al., 2013) raised [Cl^−^]_int_ to 44 mM in wild-type Bergmann glia, and TBOA blockage of both glial glutamate transporters, EAAT1 and EAAT2, raised [Cl^−^]_int_ to around 50 mM. The expression of EAATs is developmentally controlled, and such changes in EAAT expression results in a developmental chloride switch. At P8, [Cl^−^]_int_ were comparable to those of juvenile animals after blocking EAAT1 and EAAT2 with TBOA, with [Cl^−^]_int_ decreasing to adult levels between P9 and P12. These results demonstrate that EAAT anion channels are major determinants of [Cl^−^]_int_ in Bergmann glial cells (Untiet et al., 2017).

**Figure 3:**
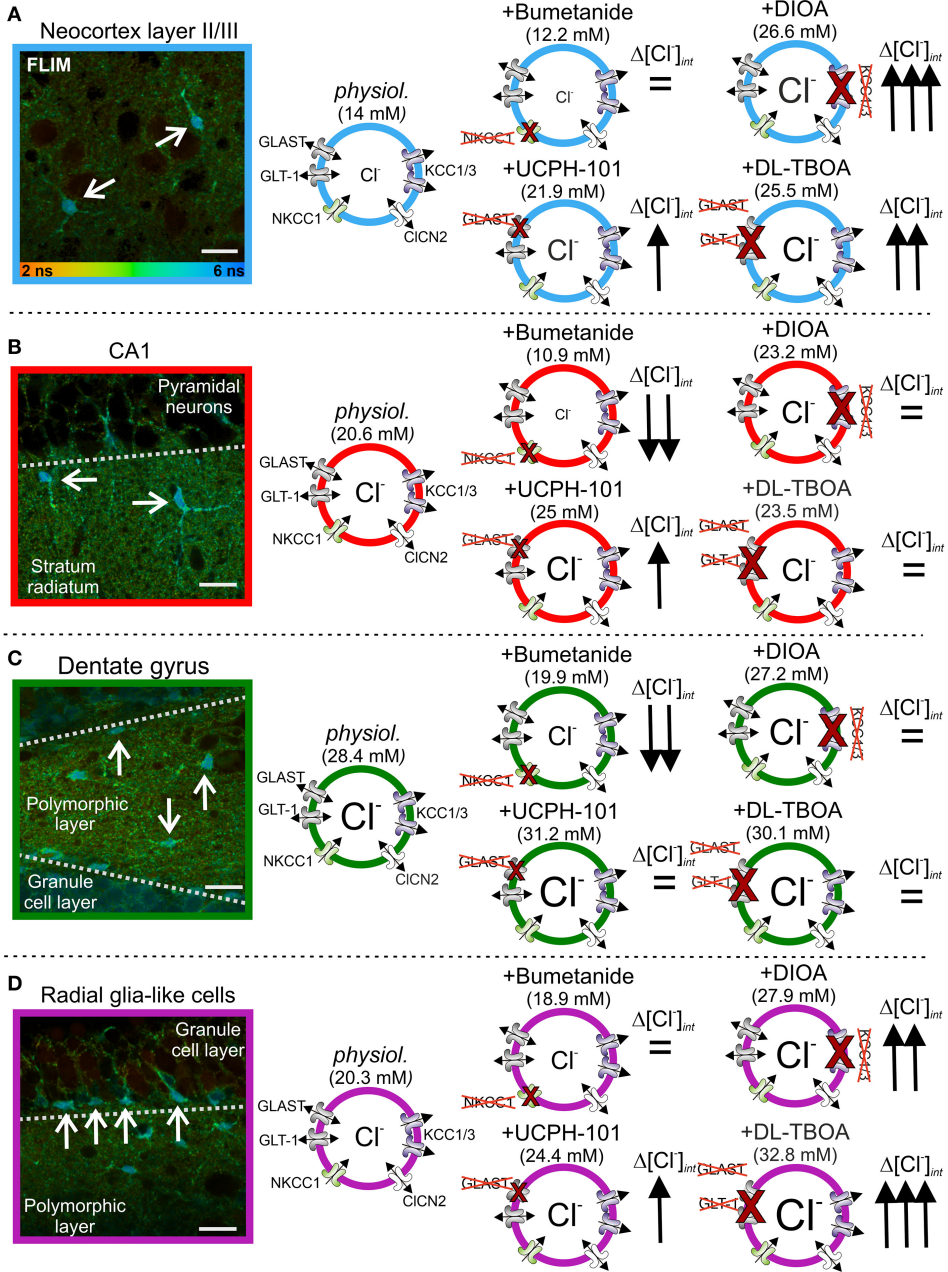
Glial [Cl^−^]_int_ varies between brain regions. **(A–D)** Fluorescence lifetime microscopy (FLIM) images of neocortical **(A)** and hippocampal **(B)** CA1 and **(C)** dentate gyrus astrocytes, as well as **(D)** radial-glia-like cells (arrows). The [Cl^−^]_int_ varies between 14 mM in cortical astrocytes and 28 mM in dentate gyrus astrocytes. Modification of [Cl^−^]_int_ by anion transport blockers illustrate that differences in anion transport protein expression cause this variability. Scale bars: 20 μm. Modified and reprinted from Engels et al. (2021), with permission.

Subsequent work on hippocampal and cortical glia revealed that resting [Cl^−^]_int_ differs even in highly similar glia types and that the contribution of EAAT anion channels in setting the glial [Cl^−^]_int_ is quite variable (Figure 3) (Engels et al., 2021). [Cl^−^]_int_ varied from 14 mM in cortical astrocytes (Figure 3A) to >20 mM in CA1 astrocytes (Figure 3B) and radial-glia-like cells (Figure 3C), and to 28 mM in dentate gyrus astrocytes (Figure 3D). The use of anion transport blockers identified differences in anion transport protein expression as the mechanistic basis of this variability. Whereas, blockage of NKCC1 by bumetanide reduced [Cl^−^]_int_ in hippocampal astrocytes (Figures 3B,C), no significant change was observed in cortical astrocytes (Figure 3A), or radial-glia-like cells (Figure 3D). Blockage of KCC1 and KCC3 or of EAAT anion channels substantially increased [Cl^−^]_int_ in cortical and CA1 astrocytes and in radial-glia-like cells (Figures 3A,B,D), but not in dentate gyrus astrocytes (Figure 3C). The experiments showed that EAAT anion channels are involved in the regulation of internal chloride concentrations of certain, but not in all glial cells.

## Glial Chloride Homeostasis in *SLC1A3*-Associated Neurological Disease

**Figure 4:**
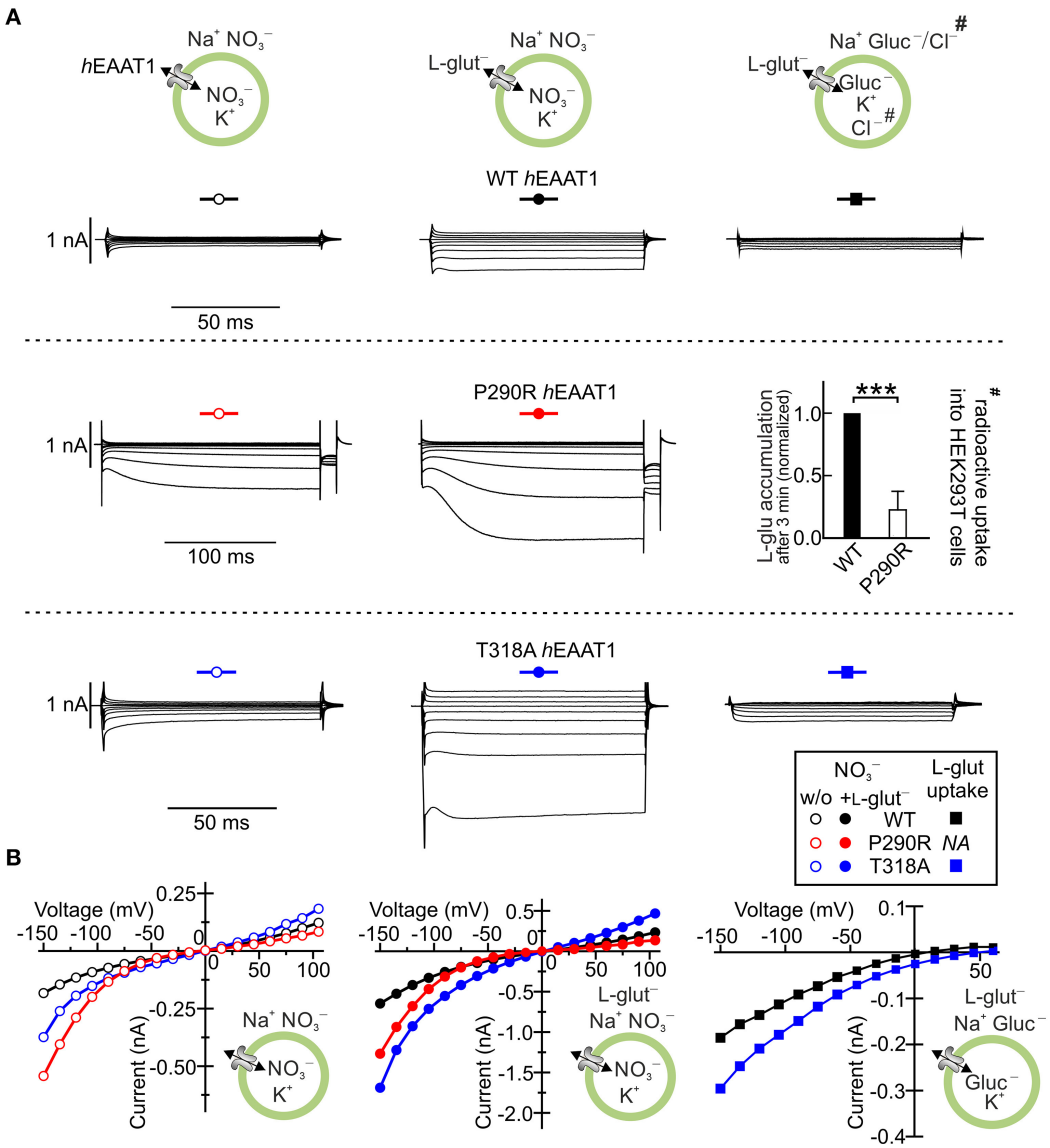
*SLC1A3* mutations associated with episodic ataxia 6 modify EAAT1 anion channels. **(A)** Representative whole-cell current recordings of human wild-type (WT) EAAT1 and episodic ataxia 6-associated EAAT1 variants P290R and T318A heterologously expressed in HEK293T cells in the absence (left) and presence (middle) of external l-glutamate. Representative L-glutamate uptake currents are shown for WT EAAT1 and the variant T318A EAAT1 (right) and the results from radioactive uptake of L-^3^[H]-glutamate^#^ into HEK293T cell lines stably expressing WT EAAT1 and the P290R EAAT1 variant (right). **(B)** Mean current–voltage relationships from whole-cell recordings of WT and variant EAAT1 proteins, as shown in **(A)**. *h*EAAT1: human EAAT1. Level of significance: ***p ≤ 0.001. Reprinted in part from Winter et al. (2012) and Chivukula et al. (2020), with permissions.

Episodic ataxias are a group of six genetic syndromes characterized by paroxysmal cerebellar incoordination and other neurological symptoms, but differing in their clinical symptoms. Episodic ataxia 6 was first reported in a 10-year-old boy with long ataxia attacks, epilepsy and cerebellar degeneration (Jen et al., 2005), but without myokymia, nystagmus, or tinnitus. The patient was heterozygous for a *SLC1A3* mutation that predicts the substitution of proline by arginine at position 290 in EAAT1. The functional consequences of P290R substitution were evaluated after heterologous expression of the mutant protein in mammalian cells and demonstrated that P290R substitution has opposing effects on the two transport functions of EAAT1: it reduces glutamate uptake (Jen et al., 2005; Winter et al., 2012) and enhances EAAT1 anion channel activity (Winter et al., 2012) (Figure 4). The neurological symptoms of the heterozygous patient were much more pronounced than the neurological phenotype of EAAT1/GLAST (the rodent EAAT1 homolog)-knockout animals (Watase et al., 1998; Stoffel et al., 2004; Miyazaki et al., 2017), suggesting that the disease is not caused by loss of function of the EAAT1 glutamate transport.

**Figure 5:**
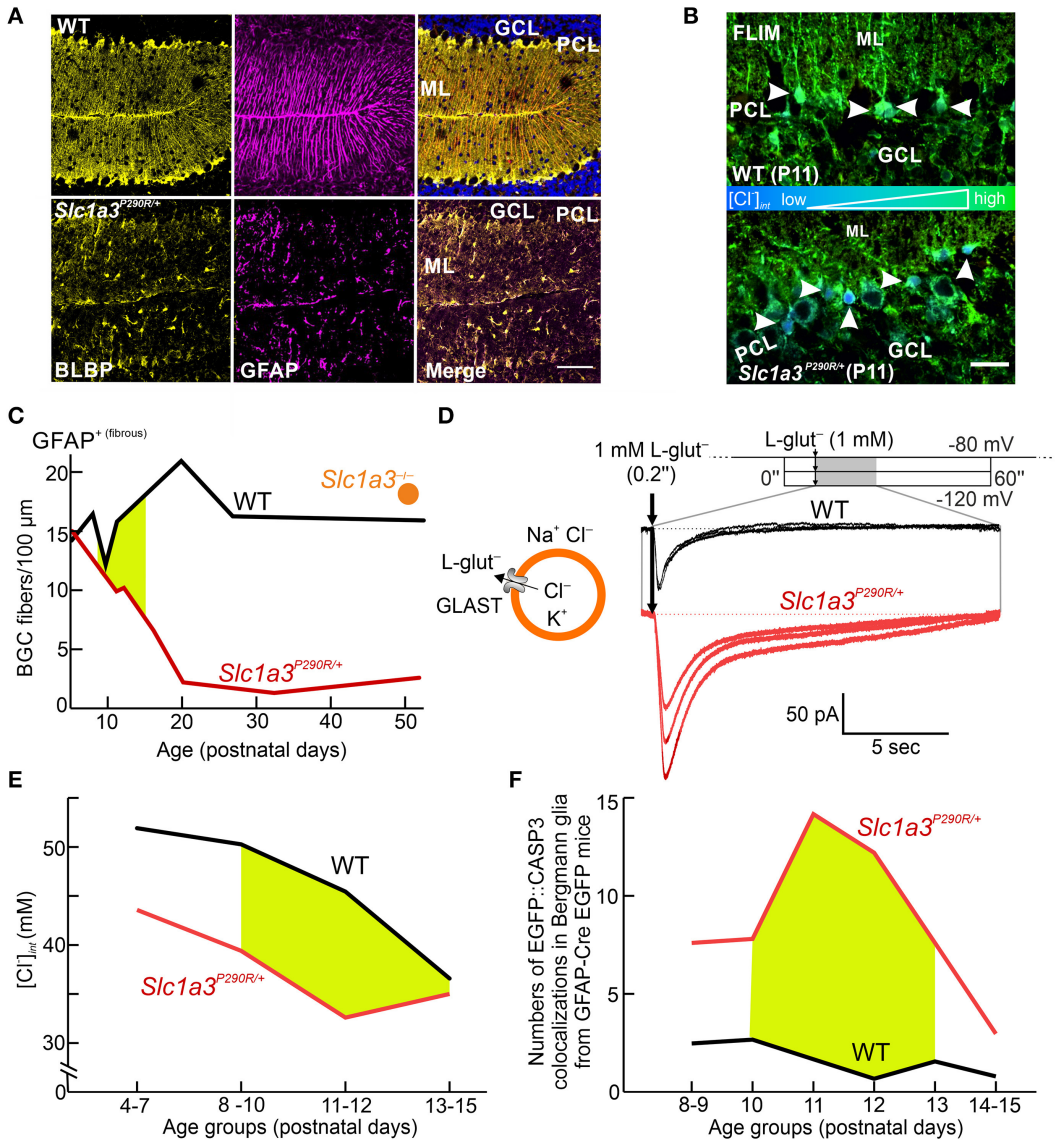
Cellular Cl^−^ depletion causes apoptosis in *Slc1a3*^*P*290*R*/+^ Bergmann glial cells. **(A)** Confocal images show the disappearance of Bergmann glial cells (BGCs) in young mice (P20) heterozygous for the P290R mutation in EAAT1/GLAST by immunostaining BGCs for brain lipid-binding protein (BLBP, yellow) and glial fibrillary acidic protein (GFAP, magenta). **(B)** FLIM imaging of cerebellar cortices from wild-type (WT) and *Slc1a3*^*P*290*R*/+^ mice indicates that P290R decreases [Cl^−^]_int_. Arrowheads depict MQAE-filled Bergmann glial cells. GCL, granule cell layer; ML, molecular layer; PCL, Purkinje cell layer. **(C)** Time course of BGC numbers in the cerebella of WT (black), *Slc1a3*^*P*290*R*/+^ (red), and *Slc1a3*^−/−^ (orange) mice shows that loss of BGCs occurs only in P290R mice—not in knockout animals. **(D)** Whole-cell patch-clamp recordings of acute brain slices from WT and *Slc1a3*^*P*290*R*/+^ animals show gain of function of *Slc1a3*^*P*290*R*/+^ anion channels in BGCs from mutant animals upon brief pulses of l-glutamate (L-glut). **(E)** [Cl^−^]_int_ is decreased in BGCs from *Slc1a3*^*P*290*R*/+^ mice at all stages of early postnatal development. The age period for BGC loss is shown in yellow. **(F)** Number of cerebellar CASP3 signals in GFAP-EGFP-expressing mice during the second week of life. Reprinted in part and modified from Kovermann et al. (2020), with permission.

A heterozygous knock-in mouse (*Slc1a3*^*P*290*R*/+^) carrying the disease-causing P290R mutation (Jen et al., 2005) showed ataxia and epilepsy, thus closely resembling the neurological symptoms of the human patient (Kovermann et al., 2020). In *Slc1a3*^*P*290*R*/+^animals, Bergmann glial cells almost completely disappeared between P10 and P20 due to apoptosis. Unaltered numbers of these cells in *Slc1a3*^−/−^ animals showed that gain of function of the EAAT1/GLAST anion channel (rather than impaired glutamate transport) causes glial apoptosis (Watase et al., 1998; Stoffel et al., 2004; Miyazaki et al., 2017; Kovermann et al., 2020) (Figures 5A,B).

Glutamate-activated Cl^−^ currents were increased in electrophysiological recordings from *Slc1a3*^*P*290*R*/+^ Bergmann glial cells (Figure 5C). Since EAAT1 contributes to chloride homeostasis in these cells, [Cl^−^]_int_ between P10 and P20 was compared in *Slc1a3*^*P*290*R*/+^ and wild-type Bergmann glia (Kovermann et al., 2020). FLIM revealed a reduction in [Cl^−^]_int_ by ~20% in *Slc1a3*^*P*290*R*/+^ Bergmann glia (Figures 5D,E), indicating an increased outward Cl^−^ flux. Apoptotic events in Bergmann glial cells were significantly increased in mutant animals over the experimental time course (Figure 5F), suggesting that increased Cl^−^ efflux through P290R EAAT1/GLAST triggers Bergmann glial cell shrinking and apoptosis (Kovermann et al., 2020). Thus, impaired glial chloride homeostasis appears to be a major pathomechanism in episodic ataxia 6.

Functional analysis of other disease-associated *SLC1A3* mutations in heterologous expression systems revealed a variety of alterations in EAAT1 function (Chivukula et al., 2020), indicating that episodic ataxia 6 is not always caused by changes in the EAAT1 anion channel function but instead involves a range of functional defects in this transporter. The mutations C186S (RefSeq: NM_004172.4: c.556T>A), A329T (NM_004172.4: c.985G>A), V393I (NM_004172.4: c.1177G>A), and R499Q (alias R454Q, XM_024446182.1: c.1361G>A) (De Vries et al., 2009; Choi et al., 2017a,b; Iwama et al., 2018) increased protein expression but decreased glutamate uptake and anion channel function in *h*EAAT1. One mutation (M128R: NM_004172.4: c.383T>G) led to complete loss of transport and channel function, accompanied by decreased protein expression. Only one of the tested mutations, threonine to alanine substitution at position 318 in *h*EAAT1 (T318A: RefSeq NM_004172.4: c.952A>G), increased anion conductance and L-glutamate uptake through increased membrane insertion by factors of 2.4 and 1.6, respectively (Chivukula et al., 2020) (Figure 4). To understand how these subtle changes in function result in cerebellar incoordination, additional disease models need to be generated and analyzed.

*SLC1A3* variants are not only associated with episodic ataxia 6. We reported a *SLC1A3* mutation in a young man with migraine with aura including hemiplegia (T387P); this mutation prevents glutamate transport by impairing K^+^ binding (Kovermann et al., 2017). A sequence variant predicting E219D in EAAT1 (RefSeq: NM_004172.4: c.657G>C) was recently associated with Tourette syndrome and hemiplegic migraine (Adamczyk et al., 2011); the variant shown to increase the surface expression of EAAT1 (Adamczyk et al., 2011). However, the E219D variant is also found in healthy individuals (gnomAD v2.1.1)—both heterozygous and homozygous—(Karczewski et al., 2020); therefore, this variant alone is unlikely to be responsible for Tourette syndrome. Gene duplication of *SLC1A3* was reported in patients with autism and attention deficit hyperactivity disorder (van Amen-Hellebrekers et al., 2016). Both *SLC1A3* duplication and the E219D variant might increase glutamate-activated anion channel currents in glial cells, and subsequent changes in anion currents or anion concentration in radial-glia-like cells might modify network formation during development, thus contributing to complex neuropsychiatric diseases. The EAAT3 variant R445W was found in a patient with symptoms of obsessive compulsive disorder who was also diagnosed with dicarboxyluria. R445 is highly conserved within vertebrate EAATs, and The R445W variant of EAAT3 has changed glutamate affinity (Bailey et al., 2011). Since this residue is critical for the anion selectivity of EAAT anion channels (Machtens et al., 2015; Cater et al., 2016), it is tempting to speculate that this variant might also affect the ion channel function of EAAT3.

## Glial Chloride Homeostasis Under Ischemic Stress

Reduced intracellular [ATP] is expected to inhibit primary active Na^+^-K^+^-ATPase and, thus, increase intracellular [Na^+^] and extracellular [K^+^]. Since such alterations stimulate NKCC and KCC transport, and also enhance EAAT anion currents *via* impaired neurotransmitter uptake, energy restrictions are likely to interfere with glial chloride homeostasis. Engels et al. recently studied glial [Cl^−^]_int_ under transient ischemic stress in acute brain slices by FLIM. Whereas, [Cl^−^]_int_ and cell volumes stayed constant during 10 min of chemical ischemia, energy depletion during blockage of NKCC1 and KCCs significantly changed [Cl^−^]_int_. Thus, metabolic stress upregulates Cl^−^ inward and outward transport; increases in Cl^−^ flux in both directions compensate for each other and keep glial [Cl^−^]_int_ constant during transient moderate ischemia (Engels et al., 2021).

Glial [Cl^−^]_int_ under both control conditions and transient energy restriction can be quantitatively described with mathematical models, in which ion transport in pre- and post-synaptic neurons and astrocytes are described with a set of differential equations (Engels et al., 2021; Kalia et al., 2021). Differences in [Cl^−^]_int_ between cortical and hippocampal astrocytes under both control conditions and energy restriction could be modeled by varying NKCC and KCC densities. The role of EAAT anion channels during ischemic chloride homeostasis was tested by modifying the TBOA-sensitive leak conductance in such simulations. This did not affect the modeling results, indicating that EAAT anion channels contribute only slightly to energy restriction-induced changes in glial [Cl^−^]_int_. Taken together, these results support the notion that glial [Cl^−^]_int_ is in dynamic equilibrium between chloride inward transport and outward flux/transport by cation-coupled transporters and EAAT anion channels and that changes in transport rates are compensated during the initial phases of transient ischemia.

## EAAT5 Improves Temporal Resolution in the Retina

**Figure 6:**
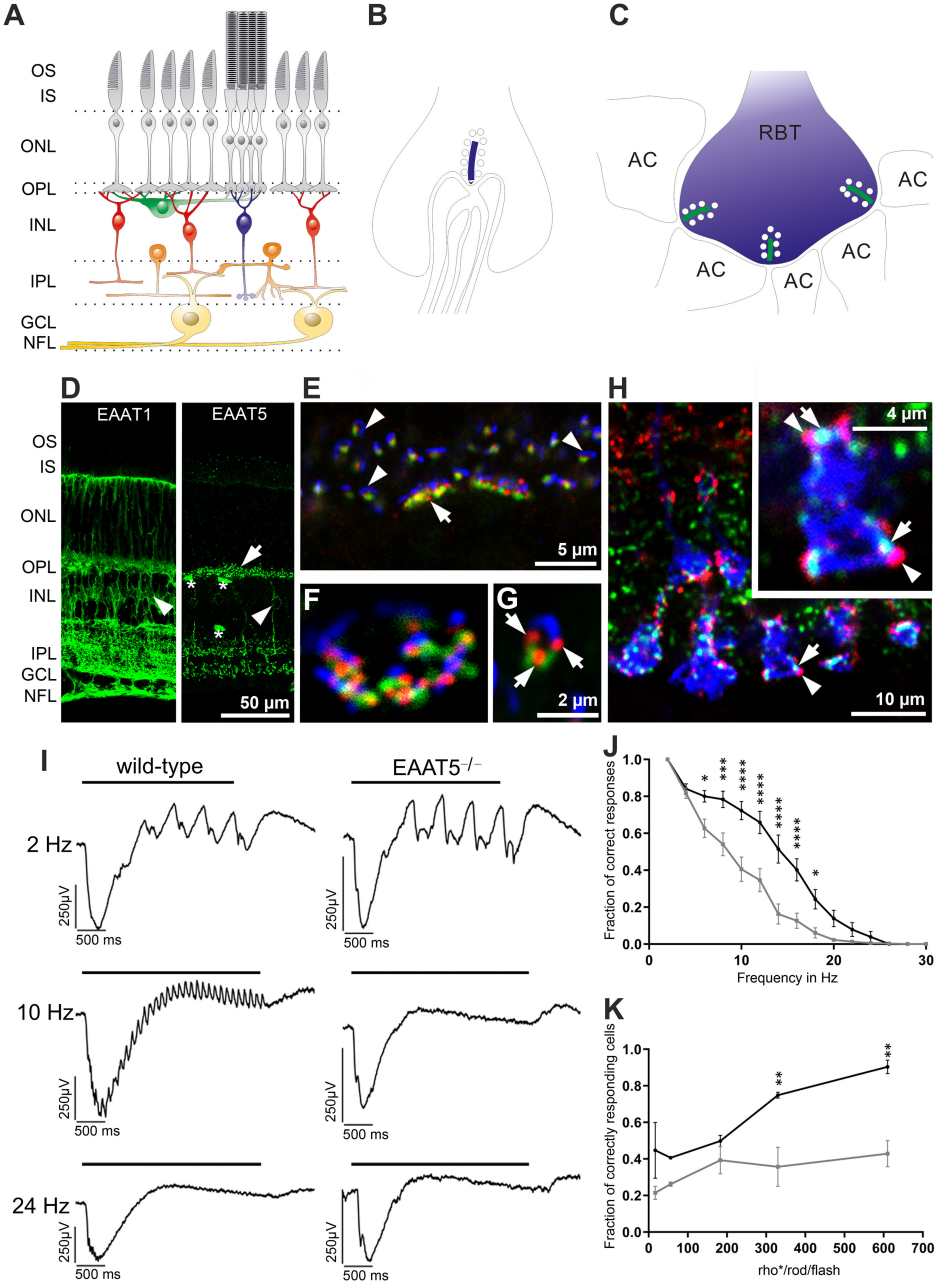
Expression and role of EAAT5 in the retina. **(A)** Schematic diagram of the retinal network (photoreceptors in gray, horizontal cells in green, cone bipolar cells in red, rod bipolar cell in blue, amacrine cells in orange, ganglion cells in yellow) with synapses formed in the outer plexiform layer (OPL) and inner plexiform layer (IPL). GCL, ganglion cell layer; INL, inner nuclear layer; IPL, inner plexiform layer; IS, inner segments; NFL, nerve fiber layer; ONL, outer nuclear layer; OPL, outer plexiform layer; OS, outer segments. **(B)** Synapse at a rod terminal with presynaptic ribbon (blue) and invaginating post-synaptic processes of bipolar and horizontal cells. **(C)** Synapses at a rod bipolar terminal (RBT) with two post-synaptic amacrine processes (AC) per ribbon (green). **(D)** Comparison of EAAT1 (left) and EAAT5 (right) expression. EAAT1 is widely expressed in retinal Müller cells (arrowhead), whose processes span the entire retina. EAAT5 is mostly found in bright puncta (arrow) in both OPL and IPL and is expressed weakly in some bipolar cell bodies (arrowhead). Asterisks mark blood vessels stained unspecifically by the secondary antibody. **(E–G)** Triple staining with antibodies against mGluR6 (green, on ON-bipolar cell dendrites), EAAT5 (red), and piccolo (blue, synaptic ribbon, glutamate-release site) in the OPL of a wild-type retina. **(E)** EAAT5 puncta are always highly associated with the glutamate-release site. Arrow: cone terminal; arrowheads: rod terminals. **(F)** Higher magnification image showing the terminal of a cone in horizontal view. **(G)** Rod terminal in side view (three EAAT5 puncta are seen at the rod terminal, arrows). **(H)** Triple staining in the IPL, showing close association of EAAT5 puncta (red, arrowheads) with the synaptic ribbons (green, arrows) on rod bipolar cell terminals (blue, colors correspond to scheme in **C**). **(I)** Recordings of local field potentials in response to flicker stimuli of different frequencies. In contrast to the wild-type retina (left), EAAT5^−/−^ retina (right) did not resolve the 10-Hz flicker stimulus. Bar represents duration of the flicker stimulus (total stimulus duration: 3 s; individual flash duration: 20 ms; 610 activated rhodopsin molecules (rho*) per rod and flash (rho*/rod/flash); mesopic conditions). **(J)** The fraction of correct responses was significantly reduced in EAAT5^−/−^ (gray curve) compared with wild-type (black curve) retina. Stimulus parameters were the same as in **(I)**. **(K)** The impact of EAAT5 on temporal resolution in ON-ganglion cells increases with stimulus intensity [indicated as activated rhodopsin molecules (rho*) per rod and flash (rho*/rod/flash)]; flicker stimulus: 12 Hz, total stimulus duration: 3 s; individual flash duration: 20 ms. Black: wild type; gray: EAAT5^−/−^. Levels of significance are: *p ≤ 0.05; **p ≤ 0.01; ***p ≤ 0.001; ****p ≤ 0.0001. Figure is modified and reprinted from Gehlen et al. (2021), with permission.

The retina is a well-layered neuronal network (Figure 6A). Glutamate is released by photoreceptors (gray) in the outer plexiform layer (OPL) and bipolar cells (red and blue) in the inner plexiform layer (IPL). Photoreceptor synapses are complex structures with invaginations that harbor several post-synaptic processes and a pre-synaptic ribbon decorated with synaptic vesicles marking the glutamate release site at each invagination (Figure 6B, rod terminal, ribbon in blue). Each bipolar cell terminal makes several output synapses with ribbons and with two postsynaptic processes per ribbon (Figure 6C, shown for a rod bipolar terminal (RBT, blue, an interneuron relaying information from rod photoreceptors), ribbons in green, AC, amacrine cell process). EAAT1 is expressed at high levels in Müller cells (Figure 6D, left) and is assumed to mediate most of the retinal glutamate uptake (Derouiche and Rauen, 1995; Rauen et al., 1996, 1998; Lehre et al., 1997; Pow and Barnett, 1999; Izumi et al., 2002; Sarthy et al., 2005). EAAT2 is expressed in photoreceptors and bipolar cells (Rauen et al., 1996, 1998; Harada et al., 1998; Rauen and Wiessner, 2000), suggesting that it has a role in glutamate reuptake and recycling in glutamatergic cells. EAAT5 expression has been described in both synaptic layers and the somata of some bipolar, amacrine, and ganglion cells, as well as in photoreceptors, including their inner segments (Pow and Barnett, 2000; Wersinger et al., 2006). However, a recent study (Gehlen et al., 2021) found that *m*EAAT5 is strongly expressed in a punctate manner (Figure 6D, right) and is closely associated with glutamate release sites (indicated by the presence of synaptic ribbons) on both rod and cone photoreceptors in the outer plexiform layer (Figures 6E–G) and rod bipolar cells in the inner plexiform layer of the mouse retina (Figure 6H). While rod bipolar cell terminals were decorated with numerous *m*EAAT5-positive puncta (Figure 6H, colors correspond to scheme in Figure 6C), the label in cone bipolar cells was less clear. Evidence for EAAT5 expression in photoreceptors and rod bipolar cells also comes from electrophysiological studies (Eliasof and Werblin, 1993; Picaud et al., 1995b; Hasegawa et al., 2006; Veruki et al., 2006; Wersinger et al., 2006; Bligard et al., 2020). The striking difference in the expression patterns of EAAT1 and EAAT5 suggests that they have different functions. Owing to its close association with the glutamate-release site, EAAT5 is perfectly located to mediate glutamate-driven negative feedback.

Recordings from isolated retinae *in vitro* revealed that *m*EAAT5 is important to achieve high temporal resolution of retinal light responses when both rods and cones are active (Gehlen et al., 2021). When recording local field potentials in response to flicker stimuli of different frequencies (Figure 6I), temporal resolution was significantly compromised in *m*EAAT5^−/−^ compared with wild-type retina (Figure 6J). A similar pattern was observed upon comparing the light responses of individual ganglion cells in the form of action potential trains. Moreover, the effect of *m*EAAT5 on temporal resolution grew stronger with increasing stimulus intensity (Figure 6K), consistent with the fact that the light-evoked modulation of glutamate release—and, hence, the impact of EAAT5—depends on the brightness of the stimulus. The effect of *m*EAAT5 deletion is in perfect agreement with the postulated role for EAAT5 at rod bipolar cell terminals, where it was shown to act as a glutamate-gated chloride channel (Veruki et al., 2006; Wersinger et al., 2006) and may be important for gain control (Bligard et al., 2020). Upon depolarization of the rod bipolar cell, glutamate release at the output synapse would activate not only the glutamate receptors on postsynaptic cells but also presynaptic EAAT5, leading to chloride influx, hyperpolarization of the cell, and consequently, reduced bipolar cell output. This negative feedback would curtail the bipolar cell response to individual flashes during repetitive stimulation and, therefore, increase temporal resolution. However, the effect of EAAT5 on glutamate buffering and reuptake in the synaptic cleft might also help to fine-tune the action of glutamate at post-synaptic cells. The synergistic action of both mechanisms might account for the increased temporal resolution in wild-type retina.

Obviously, the role of EAAT5 in photoreceptor terminals needs to be addressed in future studies. Hasegawa et al. (2006) reported that EAAT5-mediated glutamate clearance at the photoreceptor synapse is important for shaping light responses at rod–rod bipolar cell synapses in mice. However, the photoreceptor synapse is highly complex and a number of feedback mechanisms have been described. EAAT5 knockout in photoreceptors might lead to elevated levels of glutamate in the synaptic cleft, thus triggering a variety of possible mechanisms. For example, a metabotropic glutamate receptor was reported on cone terminals, and this might become activated and could affect the rate of glutamate release (Van Hook et al., 2017). EAAT5 might also affect photoreceptor output *via* its function as chloride channel by regulating [Cl^−^]_int_ at the terminal. In salamander photoreceptors, chloride dynamics in the photoreceptor terminal affect the activation properties of voltage-activated calcium channels (Thoreson et al., 2000, 2003; Thoreson and Bryson, 2004; Li et al., 2008) and, hence, synaptic transmission.

In white perch retina, a glutamate-gated chloride conductance (probably mediated by EAAT5) was also found postsynaptic to photoreceptors on dendrites of certain ON-bipolar cell types that receive a mixed input from rods and cones (Grant and Dowling, 1995). As photoreceptors are depolarized in the dark, their glutamate must hyperpolarize ON-bipolar cells. Typically, glutamate binding to mGluR6 closes TRPM1 channels in ON-bipolar cells (Koike et al., 2010; Morgans et al., 2010), leading to hyperpolarization. The ON-bipolar cells recorded by Grant and Dowling (1995) also contain a mGluR6 cascade that relays information by rod input, while cone input activates the EAAT anion conductance. Thus, in this particular ON-bipolar cell type, hyperpolarization is achieved by two functionally distinct mechanisms.

Interestingly, EAAT5 was also reported at the ribbon synapses of vestibular hair cells (Dalet et al., 2012) but not at the calyx of Held (Palmer et al., 2003), a well-studied conventional glutamatergic synapse. Compared with conventional synapses, ribbon synapses are characterized by much higher and sustained vesicular release based on graded potentials. It is, therefore, tempting to speculate that EAAT5-mediated feedback triggered by glutamate release might be a common mechanism to regulate synaptic output at ribbon synapses.

## Conclusions

Glutamate transport seems to be inseparably linked to anion channel function. Even archaebacterial EAAT homologs exhibit this curious dual function (Ryan and Mindell, 2007; Machtens et al., 2015), and not only EAAT glutamate transporters but also vesicular glutamate transporters can function as anion channels (Schenck et al., 2009; Eriksen et al., 2016). In recent years, considerable progress has been made in assigning cellular processes to EAAT anion channels in selected cell types. In glial cells, EAAT1 and EAAT2 anion channels have been shown to contribute to glial chloride homeostasis (Untiet et al., 2017; Engels et al., 2021) under normal conditions. In a human genetic disease, gain of function in EAAT anion currents causes severe neurological symptoms by impairing glial chloride homeostasis (Winter et al., 2012; Kovermann et al., 2020). In non-retinal neurons, the role of EAAT anion channels has not yet been addressed. We postulate that presynaptic EAAT2 transporters (Petr et al., 2015) may contribute to cytoplasmic [Cl^−^]_int_ in nerve terminals. As the anion channel function of vesicular glutamate transporters likely also affects glutamate accumulation in synaptic vesicles (Martineau et al., 2017), EAAT2 anion channels might contribute to setting vesicular glutamate concentrations. Additionally, presynaptic EAAT2 might fulfill similar roles to EAAT5 in the retina and mediate negative feedback in glutamate release; however, presynaptic [Cl^−^]_int_ is not currently known. The potential roles of anion channels associated with epithelial EAAT isoforms (Kanai and Hediger, 1992; Bailey et al., 2011) have not yet been investigated.

The cellular physiology and pathophysiology of EAAT anion channels are insufficiently understood, but recent progress leaves little doubt that this transport function, which was initially thought to represent transporter slippage (i.e., ion flux through accidental pore opening due to imperfect coordination of transporters during coupled transport), serves important cellular functions.

## Author Contributions

PK, ME, FM, and CF wrote the manuscript. PK and FM generated the figures. All authors approved the submitted version.

## Funding

This work was supported by the Deutsche Forschungsgemeinschaft (DFG, German Research Foundation) to CF (FA 301/13-1) as part of the Research Unit FOR 2795 (Synapses under stress) and by the German Ministry of Education and Research (E-RARE network Treat-ION, BMBF 01GM1907C to CF).

## Conflict of Interest

The authors declare that the research was conducted in the absence of any commercial or financial relationships that could be construed as a potential conflict of interest.

## Publisher's Note

All claims expressed in this article are solely those of the authors and do not necessarily represent those of their affiliated organizations, or those of the publisher, the editors and the reviewers. Any product that may be evaluated in this article, or claim that may be made by its manufacturer, is not guaranteed or endorsed by the publisher.

## References

[B1] Abrahamsen B. Schneider N. Erichsen M. N. Huynh T. H. Fahlke C. Bunch L.. (2013). Allosteric modulation of an excitatory amino acid transporter: the subtype-selective inhibitor UCPH-101 exerts sustained inhibition of EAAT1 through an intramonomeric site in the trimerization domain. J. Neurosci. 33, 1068–1087. 10.1523/JNEUROSCI.3396-12.2013 23325245 PMC6704888

[B2] Adamczyk A. Gause C. D. Sattler R. Vidensky S. Rothstein J. D. Singer H.. (2011). Genetic and functional studies of a missense variant in a glutamate transporter, SLC1A3, in Tourette syndrome. Psychiatr. Genet. 21, 90–97. 10.1097/YPG.0b013e328341a307 21233784

[B3] Arkhipova V. Trinco G. Ettema T. W. Jensen S. Slotboom D. J. Guskov A. (2019). Binding and transport of D-aspartate by the glutamate transporter homolog GltTk. Elife 8:e45286. 10.7554/eLife.45286.015 30969168 PMC6482001

[B4] Arriza J. L. Eliasof S. Kavanaugh M. P. Amara S. G. (1997). Excitatory amino acid transporter 5, a retinal glutamate transporter coupled to a chloride conductance. Proc. Natl. Acad. Sci. U.S.A. 94, 4155–4160. 10.1073/pnas.94.8.4155 9108121 PMC20584

[B5] Bailey C. G. Ryan R. M. Thoeng A. D. Ng C. King K. Vanslambrouck J. M.. (2011). Loss-of-function mutations in the glutamate transporter SLC1A1 cause human dicarboxylic aminoaciduria. J. Clin. Invest. 121, 446–453. 10.1172/JCI44474 21123949 PMC3007158

[B6] Bevensee M. O. Weed R. A. Boron W. F. (1997). Intracellular pH regulation in cultured astrocytes from rat hippocampus. I. Role Of HCO_3_?. J. Gen. Physiol. 110, 453–465. 10.1085/jgp.110.4.453 9379175 PMC2229379

[B7] Bligard G. W. DeBrecht J. Smith R. G. Lukasiewicz P. D. (2020). Light-evoked glutamate transporter EAAT5 activation coordinates with conventional feedback inhibition to control rod bipolar cell output. J. Neurophysiol. 123, 1828–1837. 10.1152/jn.00527.2019 32233906 PMC7444922

[B8] Boudker O. Ryan R. M. Yernool D. Shimamoto K. Gouaux E. (2007). Coupling substrate and ion binding to extracellular gate of a sodium-dependent aspartate transporter. Nature 445, 387–393. 10.1038/nature05455 17230192

[B9] Cater R. J. Vandenberg R. J. Ryan R. M. (2014). The domain interface of the human glutamate transporter EAAT1 mediates chloride permeation. Biophys. J. 107, 621–629. 10.1016/j.bpj.2014.05.046 25099801 PMC4129490

[B10] Cater R. J. Vandenberg R. J. Ryan R. M. (2016). Tuning the ion selectivity of glutamate transporter-associated uncoupled conductances. J. Gen. Physiol. 148, 13–24. 10.1085/jgp.201511556 27296367 PMC4924932

[B11] Chen I. Pant S. Wu Q. Cater R. J. Sobti M. Vandenberg R. J.. (2021). Glutamate transporters have a chloride channel with two hydrophobic gates. Nature 591, 327–331. 10.1038/s41586-021-03240-9 33597752 PMC7954978

[B12] Chivukula A. S. Suslova M. Kortzak D. Kovermann P. Fahlke C. (2020). Functional consequences of *SLC1A3* mutations associated with episodic ataxia 6. Hum. Mutat. 41, 1892–1905. 10.1002/humu.24089 32741053

[B13] Choi K. D. Jen J. C. Choi S. Y. Shin J. Kim H. Kim H.. (2017b). Late-onset episodic ataxia associated with *SLC1A3* mutation. J. Hum. Genet. 62, 443–446. 10.1038/jhg.2016.137 27829685

[B14] Choi K. D. Kim J. S. Kim H. J. Jung I. Jeong S. H. Lee S. H.. (2017a). Genetic variants associated with episodic ataxia in Korea. Sci. Rep. 7:13855. 10.1038/s41598-017-14254-7 29062094 PMC5653837

[B15] Crisman T. J. Qu S. Kanner B. I. Forrest L. R. (2009). Inward-facing conformation of glutamate transporters as revealed by their inverted-topology structural repeats. Proc. Natl. Acad. Sci. U.S.A. 106, 20752–20757. 10.1073/pnas.0908570106 19926849 PMC2791632

[B16] Dalet A. Bonsacquet J. Gaboyard-Niay S. Calin-Jageman I. Chidavaenzi R. L. Venteo S.. (2012). Glutamate transporters EAAT4 and EAAT5 are expressed in vestibular hair cells and calyx endings. PLoS ONE 7:e46261. 10.1371/journal.pone.0046261 23049999 PMC3457983

[B17] Danbolt N. C. (2001). Glutamate uptake. Prog. Neurobiol. 65, 1–105. 10.1016/S0301-0082(00)00067-8 11369436

[B18] De Vries B. Mamasa H. Stam A. H. Wan J. Bakker S. L. Vanmolkot K. R.. (2009). Episodic ataxia associated with EAAT1 mutation C186S affecting glutamate reuptake. Arch. Neurol. 66, 97–101. 10.1001/archneurol.2008.535 19139306

[B19] Derouiche A. Rauen T. (1995). Coincidence of L-glutamate/L-aspartate transporter (GLAST) and glutamine synthetase (GS) immunoreactions in retinal glia: evidence for coupling of GLAST and GS in transmitter clearance. J. Neurosci. Res. 42, 131–143. 10.1002/jnr.490420115 8531222

[B20] Divito C. B. Borowski J. E. Glasgow N. G. Gonzalez-Suarez A. D. Torres-Salazar D. Johnson J. W.. (2017). Glial and neuronal glutamate transporters differ in the Na^+^ requirements for activation of the substrate-independent anion conductance. Front. Mol. Neurosci. 10:150. 10.3389/fnmol.2017.00150 28611584 PMC5447070

[B21] Eliasof S. Arriza J. L. Leighton B. H. Kavanaugh M. P. Amara S. G. (1998). Excitatory amino acid transporters of the salamander retina: identification, localization, and function. J. Neurosci. 18, 698–712. 10.1523/JNEUROSCI.18-02-00698.1998 9425012 PMC6792528

[B22] Eliasof S. Werblin F. (1993). Characterization of the glutamate transporter in retinal cones of the tiger salamander. J. Neurosci. 13, 402–411. 10.1523/JNEUROSCI.13-01-00402.1993 8093715 PMC6576323

[B23] Engels M. Kalia M. Rahmati S. Petersilie L. Kovermann P. van Putten M. J. A. M.. (2021). Glial Chloride Homeostasis Under Transient Ischemic Stress. Front. Cell. Neurosci. 15:735300. 10.3389/fncel.2021.735300 34602981 PMC8481871

[B24] Eriksen J. Chang R. McGregor M. Silm K. Suzuki T. Edwards R. H. (2016). Protons regulate vesicular glutamate transporters through an allosteric mechanism. Neuron 90, 768–780. 10.1016/j.neuron.2016.03.026 27133463 PMC4886649

[B25] Fahlke C. Kortzak D. Machtens J. P. (2016). Molecular physiology of EAAT anion channels. Pflugers Arch. 468, 491–502. 10.1007/s00424-015-1768-3 26687113

[B26] Fairman W. A. Vandenberg R. J. Arriza J. L. Kavanaugh M. P. Amara S. G. (1995). An excitatory amino-acid transporter with properties of a ligand-gated chloride channel. Nature 375, 599–603. 10.1038/375599a0 7791878

[B27] Futamachi K. J. Pedley T. A. (1976). Glial cells and extracellular potassium: their relationship in mammalian cortex. Brain Res. 109, 311–322. 10.1016/0006-8993(76)90532-1 1276917

[B28] Gameiro A. Braams S. Rauen T. Grewer C. (2011). The discovery of slowness: Low-capacity transport and slow anion channel gating by the glutamate transporter EAAT5. Biophys. J. 100, 2623–2632. 10.1016/j.bpj.2011.04.034 21641307 PMC3117175

[B29] Gehlen J. Aretzweiler C. Mataruga A. Fahlke C. Müller F. (2021). Excitatory amino acid transporter EAAT5 improves temporal resolution in the retina. eNeuro 8, 1–15. 10.1523/ENEURO.0406-21.2021 34772693 PMC8670604

[B30] Gendreau S. Voswinkel S. Torres-Salazar D. Lang N. Heidtmann H. Detro-Dassen S.. (2004). A trimeric quaternary structure is conserved in bacterial and human glutamate transporters. J. Biol. Chem. 279, 39505–39512. 10.1074/jbc.M408038200 15265858

[B31] Grant G. B. Dowling J. E. (1995). A glutamate-activated chloride current in cone-driven ON bipolar cells of the white perch retina. J. Neurosci. 15(5 Pt 2), 3852–3862. 10.1523/JNEUROSCI.15-05-03852.1995 7538566 PMC6578192

[B32] Grewer C. Balani P. Weidenfeller C. Bartusel T. Tao Z. Rauen T. (2005). Individual subunits of the glutamate transporter EAAC1 homotrimer function independently of each other. Biochemistry 44, 11913–11923. 10.1021/bi050987n 16128593 PMC2459315

[B33] Grewer C. Gameiro A. Zhang Z. Tao Z. Braams S. Rauen T. (2008). Glutamate forward and reverse transport: from molecular mechanism to transporter-mediated release after ischemia. IUBMB Life 60, 609–619. 10.1002/iub.98 18543277 PMC2632779

[B34] Guskov A. Jensen S. Faustino I. Marrink S. J. Slotboom D. J. (2016). Coupled binding mechanism of three sodium ions and aspartate in the glutamate transporter homologue GltTk. Nat. Commun. 7:13420. 10.1038/ncomms13420 27830699 PMC5110648

[B35] Harada T. Harada C. Watanabe M. Inoue Y. Sakagawa T. Nakayama N.. (1998). Functions of the two glutamate transporters GLAST and GLT-1 in the retina. Proc. Natl. Acad. Sci. U.S.A. 95, 4663–4666. 10.1073/pnas.95.8.4663 9539795 PMC22547

[B36] Hasegawa J. Obara T. Tanaka K. Tachibana M. (2006). High-density presynaptic transporters are required for glutamate removal from the first visual synapse. Neuron 50, 63–74. 10.1016/j.neuron.2006.02.022 16600856

[B37] Iwama K. Iwata A. Shiina M. Mitsuhashi S. Miyataka S. Takata A.. (2018). A novel mutation in *SLC1A3* causes episodic ataxia. J. Hum. Genet. 63, 207–211. 10.1038/s10038-017-0365-z 29208948

[B38] Izumi Y. Shimamoto K. Benz A. M. Hammerman S. B. Olney J. W. Zorumski C. F. (2002). Glutamate transporters and retinal excitotoxicity. Glia 39, 58–68. 10.1002/glia.10082 12112376

[B39] Jen J. C. Wan J. Palos T. P. Howard B. D. Baloh R. W. (2005). Mutation in the glutamate transporter EAAT1 causes episodic ataxia, hemiplegia, and seizures. Neurology 65, 529–534. 10.1212/01.WNL.0000172638.58172.5a 16116111

[B40] Jensen S. Guskov A. Rempel S. Hanelt I. Slotboom D. J. (2013). Crystal structure of a substrate-free aspartate transporter. Nat. Struct. Mol. Biol. 20, 1224–1226. 10.1038/nsmb.2663 24013209

[B41] Kalia M. Meijer H. G. E. Van Gils S. A. van Putten M. J. Rose C. R. (2021). Ion dynamics at the tripartite synapse. PLoS Comput. Biol. 17:e1009019. 10.1371/journal.pcbi.1009019 34143772 PMC8244923

[B42] Kanai Y. Hediger M. A. (1992). Primary structure and functional characterization of a high-affinity glutamate transporter. Nature 360, 467–471. 10.1038/360467a0 1280334

[B43] Kanner B. I. (2006). Structure and function of sodium-coupled GABA and glutamate transporters. J. Membr. Biol. 213, 89–100. 10.1007/s00232-006-0877-5 17417704

[B44] Karczewski K. J. Francioli L. C. Tiao G. Cummings B. B. Alfoldi J. Wang Q.. (2020). The mutational constraint spectrum quantified from variation in 141,456 humans. Nature 581, 434–443. 10.1038/s41586-020-2308-7 32461654 PMC7334197

[B45] Kettenmann H. Backus K. H. Schachner M. (1987). γ-Aminobutyric acid opens Cl-channels in cultured astrocytes. Brain Res. 404, 1–9. 10.1016/0006-8993(87)91349-7 2436707

[B46] Kimelberg H. K. (1981). Active accumulation and exchange transport of chloride in astroglial cells in culture. Biochim. Biophys. Acta 646, 179–184. 10.1016/0005-2736(81)90285-6 6268162

[B47] Koch H. P. Brown R. L. Larsson H. P. (2007). The glutamate-activated anion conductance in excitatory amino acid transporters is gated independently by the individual subunits. J. Neurosci. 27, 2943–2947. 10.1523/JNEUROSCI.0118-07.2007 17360917 PMC2435202

[B48] Koike C. Obara T. Uriu Y. Numata T. Sanuki R. Miyata K.. (2010). TRPM1 is a component of the retinal ON bipolar cell transduction channel in the mGluR6 cascade. Proc. Natl. Acad. Sci. U.S.A. 107, 332–337. 10.1073/pnas.0912730107 19966281 PMC2806705

[B49] Kolen B. Kortzak D. Franzen A. Fahlke C. (2020). An amino-terminal point mutation increases EAAT2 anion currents without affecting glutamate transport rates. J. Biol. Chem. 295, 14936–14947. 10.1074/jbc.RA120.013704 32820048 PMC7606670

[B50] Kortzak D. Alleva C. Weyand I. Ewers D. Zimmermann M. I. Franzen A.. (2019). Allosteric gate modulation confers K^+^ coupling in glutamate transporters. EMBO J. 38:e101468. 10.15252/embj.2019101468 31506973 PMC6769379

[B51] Kovermann P. Hessel M. Kortzak D. Jen J. C. Koch J. Fahlke C.. (2017). Impaired K^+^ binding to glial glutamate transporter EAAT1 in migraine. Sci. Rep. 7:13913. 10.1038/s41598-017-14176-4 29066757 PMC5654970

[B52] Kovermann P. Machtens J. P. Ewers D. Fahlke C. (2010). A conserved aspartate determines pore properties of anion channels associated with excitatory amino acid transporter 4 (EAAT4). J. Biol. Chem. 285, 23676–23686. 10.1074/jbc.M110.126557 20519505 PMC2911312

[B53] Kovermann P. Untiet V. Kolobkova Y. Engels M. Baader S. Schilling K.. (2020). Increased glutamate transporter-associated anion currents cause glial apoptosis in episodic ataxia 6. Brain Commun. 2:fcaa022. 10.1093/braincomms/fcaa022 32954283 PMC7425361

[B54] Kutzner C. Grubmuller H. de Groot B. L. Zachariae U. (2011). Computational electrophysiology: the molecular dynamics of ion channel permeation and selectivity in atomistic detail. Biophys. J. 101, 809–817. 10.1016/j.bpj.2011.06.010 21843471 PMC3175076

[B55] Larsson H. P. Picaud S. A. Werblin F. S. Lecar H. (1996). Noise analysis of the glutamate-activated current in photoreceptors. Biophys. J. 70, 733–742. 10.1016/S0006-3495(96)79613-3 8789090 PMC1224973

[B56] Leary G. P. Stone E. F. Holley D. C. Kavanaugh M. P. (2007). The glutamate and chloride permeation pathways are colocalized in individual neuronal glutamate transporter subunits. J. Neurosci. 27, 2938–2942. 10.1523/JNEUROSCI.4851-06.2007 17360916 PMC6672579

[B57] Lehre K. P. Davanger S. Danbolt N. C. (1997). Localization of the glutamate transporter protein GLAST in rat retina. Brain Res. 744, 129–137. 10.1016/S0006-8993(96)01022-0 9030421

[B58] Leinenweber A. Machtens J. P. Begemann B. Fahlke C. (2011). Regulation of glial glutamate transporters by C-terminal domains. J. Biol. Chem. 286, 1927–1937. 10.1074/jbc.M110.153486 21097502 PMC3023489

[B59] Li B. McKernan K. Shen W. (2008). Spatial and temporal distribution patterns of Na-K-2Cl cotransporter in adult and developing mouse retinas. Vis. Neurosci. 25, 109–123. 10.1017/S0952523808080164 18442435 PMC5531596

[B60] Lothman E. W. Somjen G. G. (1975). Extracellular potassium activity, intracellular and extracellular potential responses in the spinal cord. J. Physiol. 252, 115–136. 10.1113/jphysiol.1975.sp011137 1202194 PMC1348471

[B61] Machtens J. P. Kortzak D. Lansche C. Leinenweber A. Kilian P. Begemann B.. (2015). Mechanisms of anion conduction by coupled glutamate transporters. Cell 160, 542–553. 10.1016/j.cell.2014.12.035 25635461

[B62] Machtens J. P. Kovermann P. Fahlke C. (2011). Substrate-dependent gating of anion channels associated with excitatory amino acid transporter 4. J. Biol. Chem. 286, 23780–23788. 10.1074/jbc.M110.207514 21572047 PMC3129159

[B63] Martineau M. Guzman R. E. Fahlke C. Klingauf J. (2017). VGLUT1 functions as a glutamate/proton exchanger with chloride channel activity in hippocampal glutamatergic synapses. Nat. Commun. 8:2279. 10.1038/s41467-017-02367-6 29273736 PMC5741633

[B64] Mim C. Balani P. Rauen T. Grewer C. (2005). The glutamate transporter subtypes EAAT4 and EAATs 1-3 transport glutamate with dramatically different kinetics and voltage dependence but share a common uptake mechanism. J. Gen. Physiol. 126, 571–589. 10.1085/jgp.200509365 16316976 PMC2266596

[B65] Miyazaki T. Yamasaki M. Hashimoto K. Kohda K. Yuzaki M. Shimamoto K.. (2017). Glutamate transporter GLAST controls synaptic wrapping by Bergmann glia and ensures proper wiring of Purkinje cells. Proc. Natl. Acad. Sci. U.S.A. 114, 7438–7443. 10.1073/pnas.1617330114 28655840 PMC5514701

[B66] Morgans C. W. Brown R. L. Duvoisin R. M. (2010). TRPM1: the endpoint of the mGluR6 signal transduction cascade in retinal ON-bipolar cells. Bioessays 32, 609–614. 10.1002/bies.200900198 20544736 PMC4238414

[B67] Nothmann D. Leinenweber A. Torres-Salazar D. Kovermann P. Hotzy J. Gameiro A.. (2011). Hetero-oligomerization of neuronal glutamate transporters. J. Biol. Chem. 286, 3935–3943. 10.1074/jbc.M110.187492 21127051 PMC3030394

[B68] Palmer M. J. Taschenberger H. Hull C. Tremere L. von Gersdorff H. (2003). Synaptic activation of presynaptic glutamate transporter currents in nerve terminals. J. Neurosci. 23, 4831–4841. 10.1523/JNEUROSCI.23-12-04831.2003 12832505 PMC3586552

[B69] Petr G. T. Sun Y. Frederick N. M. Zhou Y. Dhamne S. C. Hameed M. Q.. (2015). Conditional deletion of the glutamate transporter GLT-1 reveals that astrocytic GLT-1 protects against fatal epilepsy while neuronal GLT-1 contributes significantly to glutamate uptake into synaptosomes. J. Neurosci. 35, 5187–5201. 10.1523/JNEUROSCI.4255-14.2015 25834045 PMC4380995

[B70] Picaud S. A. Larsson H. P. Grant G. B. Lecar H. Werblin F. S. (1995b). Glutamate-gated chloride channel with glutamate-transporter-like properties in cone photoreceptors of the tiger salamander. J. Neurophysiol. 74, 1760–1771. 10.1152/jn.1995.74.4.1760 8989410

[B71] Picaud S. A. Larsson H. P. Wellis D. P. Lecar H. Werblin F. (1995a). Cone photoreceptors respond to their own glutamate release in the tiger salamander. Proc. Natl. Acad. Sci. U.S.A. 92, 9417–9421. 10.1073/pnas.92.20.9417 7568144 PMC40996

[B72] Pines G. Danbolt N. C. Bjoras M. Zhang Y. Bendahan A. Eide L.. (1992). Cloning and expression of a rat brain L-glutamate transporter. Nature 360, 464–467. 10.1038/360464a0 1448170

[B73] Pow D. V. Barnett N. L. (1999). Changing patterns of spatial buffering of glutamate in developing rat retinae are mediated by the Muller cell glutamate transporter GLAST. Cell Tissue Res. 297, 57–66. 10.1007/s004410051333 10398883

[B74] Pow D. V. Barnett N. L. (2000). Developmental expression of excitatory amino acid transporter 5: a photoreceptor and bipolar cell glutamate transporter in rat retina. Neurosci. Lett. 280, 21–24. 10.1016/S0304-3940(99)00988-X 10696802

[B75] Rauen T. Rothstein J. D. Wassle H. (1996). Differential expression of three glutamate transporter subtypes in the rat retina. Cell Tissue Res. 286, 325–336. 10.1007/s004410050702 8929335

[B76] Rauen T. Taylor W. R. Kuhlbrodt K. Wiessner M. (1998). High-affinity glutamate transporters in the rat retina: a major role of the glial glutamate transporter GLAST-1 in transmitter clearance. Cell Tissue Res. 291, 19–31. 10.1007/s004410050976 9394040

[B77] Rauen T. Wiessner M. (2000). Fine tuning of glutamate uptake and degradation in glial cells: common transcriptional regulation of GLAST1 and GS. Neurochem. Int. 37, 179–189. 10.1016/S0197-0186(00)00021-8 10812203

[B78] Reyes N. Ginter C. Boudker O. (2009). Transport mechanism of a bacterial homologue of glutamate transporters. Nature 462, 880–885. 10.1038/nature08616 19924125 PMC2934767

[B79] Ryan R. M. Mindell J. A. (2007). The uncoupled chloride conductance of a bacterial glutamate transporter homolog. Nat. Struct. Mol. Biol. 14, 365–371. 10.1038/nsmb1230 17435767

[B80] Ryan R. M. Mitrovic A. D. Vandenberg R. J. (2004). The chloride permeation pathway of a glutamate transporter and its proximity to the glutamate translocation pathway. J. Biol. Chem. 279, 20742–20751. 10.1074/jbc.M304433200 14982939

[B81] Sarthy V. P. Pignataro L. Pannicke T. Weick M. Reichenbach A. Harada T.. (2005). Glutamate transport by retinal Muller cells in glutamate/aspartate transporter-knockout mice. Glia 49, 184–196. 10.1002/glia.20097 15390100

[B82] Schenck S. Wojcik S. M. Brose N. Takamori S. (2009). A chloride conductance in VGLUT1 underlies maximal glutamate loading into synaptic vesicles. Nat. Neurosci. 12, 156–162. 10.1038/nn.2248 19169251

[B83] Schneider N. Cordeiro S. Machtens J. P. Braams S. Rauen T. Fahlke C. (2014). Functional properties of the retinal glutamate transporters GLT-1c and EAAT5. J. Biol. Chem. 289:1815. 10.1074/jbc.M113.517177 24307171 PMC3894357

[B84] Stoffel W. Korner R. Wachtmann D. Keller B. U. (2004). Functional analysis of glutamate transporters in excitatory synaptic transmission of GLAST1 and GLAST1/EAAC1 deficient mice. Brain Res. Mol. Brain Res. 128, 170–181. 10.1016/j.molbrainres.2004.06.026 15363892

[B85] Storck T. Schulte S. Hofmann K. Stoffel W. (1992). Structure, expression, and functional analysis of a Na^+^-dependent glutamate/aspartate transporter from rat brain. Proc. Natl. Acad. Sci. U.S.A. 89, 10955–10959. 10.1073/pnas.89.22.10955 1279699 PMC50461

[B86] Tao Z. Zhang Z. Grewer C. (2006). Neutralization of the aspartic acid residue Asp-367, but not Asp-454, inhibits binding of Na^+^ to the glutamate-free form and cycling of the glutamate transporter EAAC1. J. Biol. Chem. 281, 10263–10272. 10.1074/jbc.M510739200 16478724 PMC2430067

[B87] Thoreson W. B. Bryson E. J. (2004). Chloride equilibrium potential in salamander cones. BMC Neurosci. 5:53. 10.1186/1471-2202-5-53 15579212 PMC539262

[B88] Thoreson W. B. Bryson E. J. Rabl K. (2003). Reciprocal interactions between calcium and chloride in rod photoreceptors. J. Neurophysiol. 90, 1747–1753. 10.1152/jn.00932.2002 12724369

[B89] Thoreson W. B. Nitzan R. Miller R. F. (2000). Chloride efflux inhibits single calcium channel open probability in vertebrate photoreceptors: chloride imaging and cell-attached patch-clamp recordings. Vis. Neurosci. 17, 197–206. 10.1017/S0952523800172025 10824674

[B90] Torres-Salazar D. Fahlke C. (2007). Neuronal glutamate transporters vary in substrate transport rate but not in unitary anion channel conductance. J. Biol. Chem. 282, 34719–34726. 10.1074/jbc.M704118200 17908688

[B91] Untiet V. Kovermann P. Gerkau N. J. Gensch T. Rose C. R. Fahlke C. (2017). Glutamate transporter-associated anion channels adjust intracellular chloride concentrations during glial maturation. Glia 65, 388–400. 10.1002/glia.23098 27859594

[B92] van Amen-Hellebrekers C. J. Jansen S. Pfundt R. Schuurs-Hoeijmakers J. H. Koolen D. A. Marcelis C. L.. (2016). Duplications of *SLC1A3*: Associated with ADHD and autism. Eur. J. Med. Genet. 59, 373–376. 10.1016/j.ejmg.2016.06.003 27296938

[B93] Van Hook M. J. Babai N. Zurawski Z. Yim Y. Y. Hamm H. E. Thoreson W. B. (2017). A presynaptic group III mGluR recruits Gβγ/SNARE interactions to inhibit synaptic transmission by cone photoreceptors in the vertebrate retina. J. Neurosci. 37, 4618–4634. 10.1523/JNEUROSCI.2948-16.2017 28363980 PMC5413191

[B94] Verdon G. Boudker O. (2012). Crystal structure of an asymmetric trimer of a bacterial glutamate transporter homolog. Nat. Struct. Mol. Biol. 19, 355–357. 10.1038/nsmb.2233 22343718 PMC3633560

[B95] Verdon G. Oh S. Serio R. N. Boudker O. (2014). Coupled ion binding and structural transitions along the transport cycle of glutamate transporters. Elife 3:e02283. 10.7554/eLife.02283.029 24842876 PMC4051121

[B96] Veruki M. L. Morkve S. H. Hartveit E. (2006). Activation of a presynaptic glutamate transporter regulates synaptic transmission through electrical signaling. Nat. Neurosci. 9, 1388–1396. 10.1038/nn1793 17041592

[B97] Wadiche J. I. Amara S. G. Kavanaugh M. P. (1995). Ion fluxes associated with excitatory amino acid transport. Neuron 15, 721–728. 10.1016/0896-6273(95)90159-0 7546750

[B98] Wadiche J. I. Kavanaugh M. P. (1998). Macroscopic and microscopic properties of a cloned glutamate transporter/chloride channel. J. Neurosci. 18, 7650–7661. 10.1523/JNEUROSCI.18-19-07650.1998 9742136 PMC6793006

[B99] Walz W. (2002). Chloride/anion channels in glial cell membranes. Glia 40, 1–10. 10.1002/glia.10125 12237839

[B100] Watase K. Hashimoto K. Kano M. Yamada K. Watanabe M. Inoue Y.. (1998). Motor discoordination and increased susceptibility to cerebellar injury in GLAST mutant mice. Eur. J. Neurosci. 10, 976–988. 10.1046/j.1460-9568.1998.00108.x 9753165

[B101] Wersinger E. Schwab Y. Sahel J. A. Rendon A. Pow D. V. Picaud S.. (2006). The glutamate transporter EAAT5 works as a presynaptic receptor in mouse rod bipolar cells. J. Physiol. 577(Pt 1), 221–234. 10.1113/jphysiol.2006.118281 16973698 PMC2000664

[B102] Winter N. Kovermann P. Fahlke C. (2012). A point mutation associated with episodic ataxia 6 increases glutamate transporter anion currents. Brain 135(Pt 11), 3416–3425. 10.1093/brain/aws255 23107647

[B103] Yernool D. Boudker O. Folta-Stogniew E. Gouaux E. (2003). Trimeric subunit stoichiometry of the glutamate transporters from *Bacillus caldotenax* and *Bacillus stearothermophilus*. Biochemistry 42, 12981–12988. 10.1021/bi030161q 14596613

[B104] Yernool D. Boudker O. Jin Y. Gouaux E. (2004). Structure of a glutamate transporter homologue from *Pyrococcus horikoshii*. Nature 431, 811–818. 10.1038/nature03018 15483603

